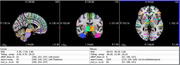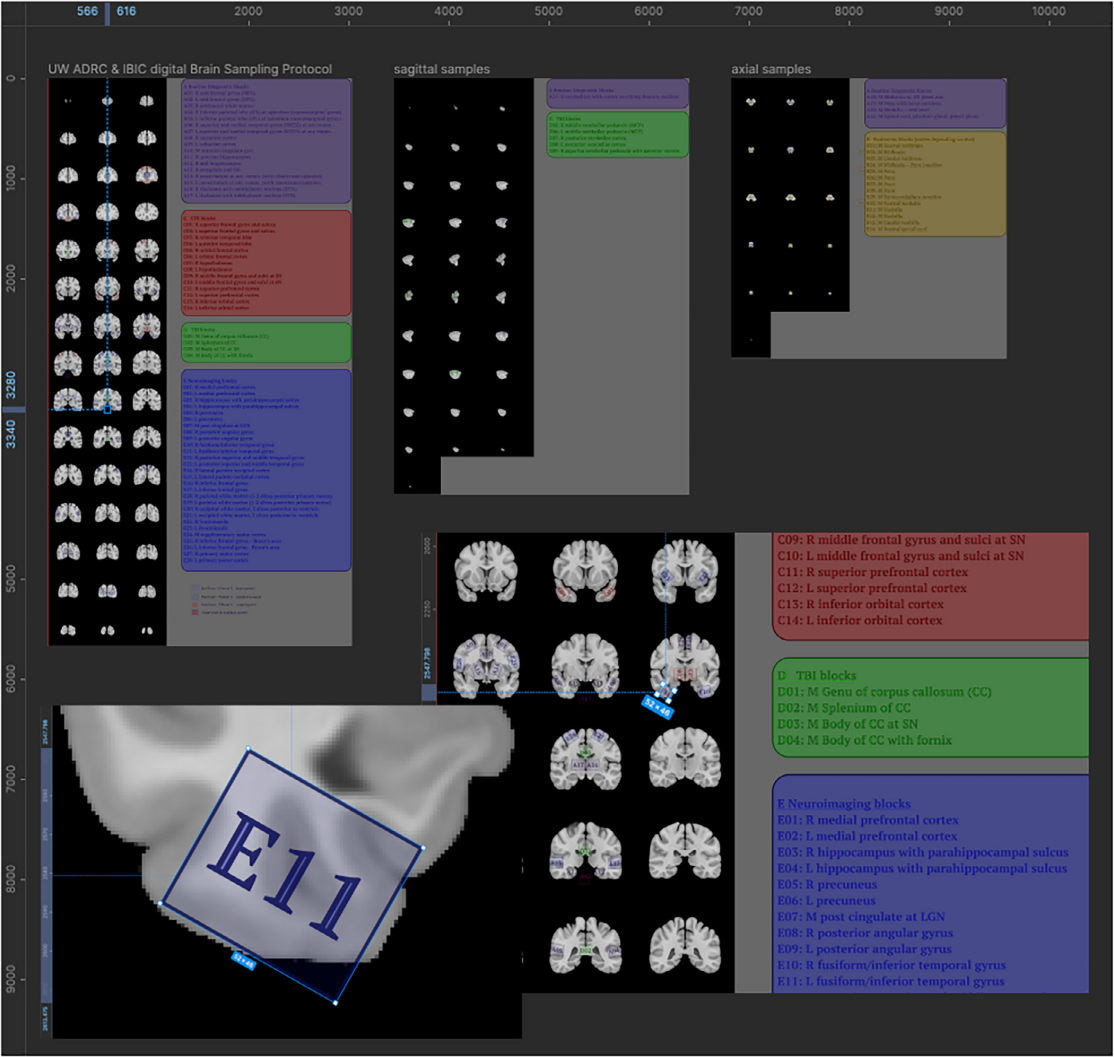# A digital brain sampling protocol atlas to simplify and accelerate biorepository tissue requests

**DOI:** 10.1002/alz.093542

**Published:** 2025-01-03

**Authors:** Jason Webster, Ali Shojaie, Yiqin Alicia Shen, Tung Le, Christine MacDonald, Caitlin S Latimer, C Dirk Keene, Thomas J Grabowski

**Affiliations:** ^1^ University of Washington, Seattle, WA USA; ^2^ University of Washington Department of Neurological Surgery, Seattle, WA USA; ^3^ University of Washington, School of Medicine, Seattle, WA USA; ^4^ Department of Laboratory Medicine and Pathology, University of Washington, Seattle, WA USA

## Abstract

**Background:**

The structural, cellular, and biomolecular research necessary for a mechanistic understanding of Alzheimer’s Disease (AD) relies on brain tissue collected according to a brain sampling protocol (BSP) and preserved in biorepositories. Such research involves discipline‐specific terminology such as cytoarchitectonic domains, brain network nodes, etc. This specificity can result in iterative, time‐consuming, and error‐prone request processes. The digital BSP atlas will streamline this process and offer resources for reference, visualization, training, and refinement of BSPs.

**Method:**

**Virtual Neuropathology** was performed by a neuropathology technician and Board‐certified neuropathologist in Figma, a collaborative cloud‐based platform with an intuitive dynamic interface, in which metric properties of the brain slices and samples from the University of Washington BioRepository and Integrated Neuropathology (BRaIN) Laboratory BSP were precisely replicated. Virtual brain slices were taken every 4mm from the Montreal Neurological Institute and International Consortium on Brain Mapping 2009b Nonlinear Asymmetric Brain Template, which has 8x higher resolution than typical brain templates. Data were exported in Scalable Vector Graphics and processed in python.

**Results:**

**The Digital BSP Atlas** contains volumetric labels in MNI 2009b space for the routinely collected samples in the BRaIN lab BSP and is stored using standard neuroinformatics conventions for use with freely available software such as FMRIB’s Software Library (FSL) and FreeSurfer.

For each sample, volume of brain regions defined by anatomical regions, brain areas, white matter pathways, subcortical structure, cytoarchitectonic domains, functional regions, or functional connectivity network nodes was calculated as the overlap with labels from a range of digital atlases which registered to MNI 2009b space.

**Conclusion:**

The digital BSP atlas provides a quantitative representation of the BSP in a standard space which will accelerating the process of requesting brain tissue, allows for the refinement of protocols, and the coordination of neuroimaging information with neuropathology sample. Future directions of this work include customizable visualizations, training software, and website backend to identify corresponding samples from free‐form text with arbitrary neuroscience terms. This research is part of a pipeline to facilitate mechanistic understanding of AD by enhancing the precision and accuracy of neuropathology sampling and robust registration to premortem neuroimaging data even for late‐stage AD participants.